# Supplementary material for: Comprehensive antibiotic-linked mutation assessment by resistance mutation sequencing (RM-seq)
Source: Genome Med. 2018 Aug 31;10:63. doi: 10.1186/s13073-018-0572-z (PMC6117896; doi:10.1186/s13073-018-0572-z)
Supplement: Supplementary file 2 — Figure S1. 3D model of S. aureus RpoB protein in complex with rifampicin. Figure S2. Prediction of the number of consensus reads at different sequencing depths. (PDF 648 kb) [file 13073_2018_572_MOESM2_ESM.pdf]

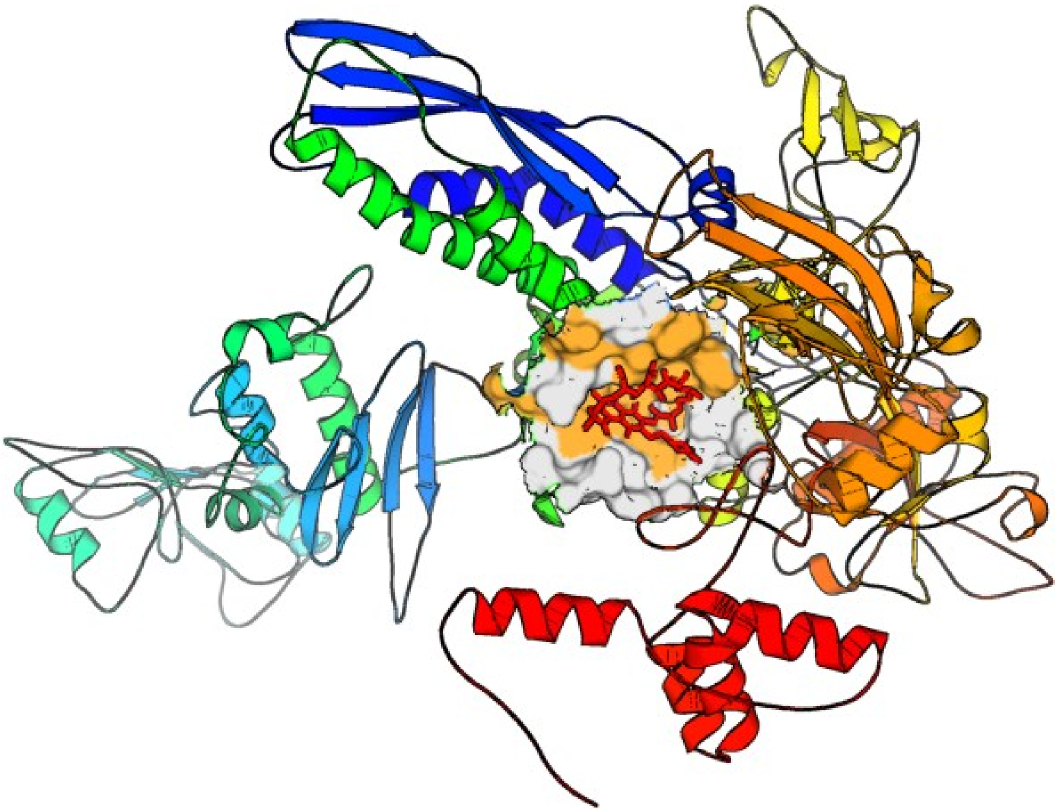

Figure S1: 3D model of *S. aureus* RpoB in complex with rifampicin. Rifampicin molecule is coloured in red. The RpoB residue surface a distance of less than 10Å are coloured in white, residues associated with rifampicin resistance by RM-seq are coloured in orange. S. aureus NRS384 WT RpoB protein structure was modelled on the Swiss-model server (https://swissmodel.expasy.org) using Escherichia coli RNA polymerase and rifampicin complex structure (5UAC). The structure model was visualised using PyMOL software.

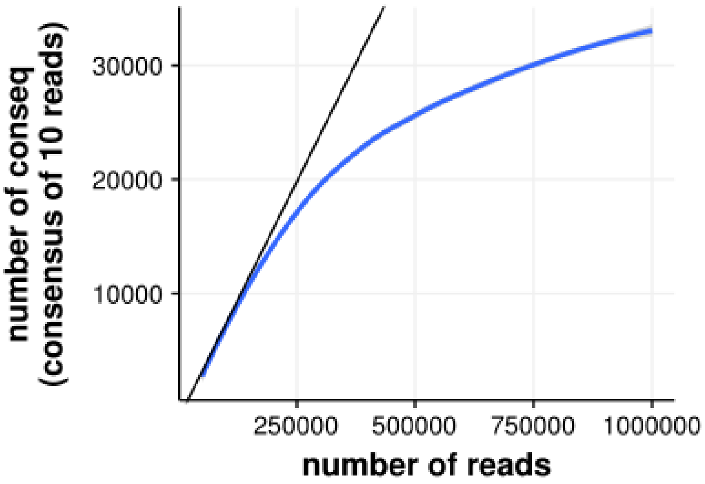

Figure S2: Prediction of the number of consensus reads at different sequencing depths. The graph shows the number of error-corrected consensus reads generated from at least 10 reads at different sequencing depths. The figure has been generated by subsampling (50,000 to 1,000,000 paired reads with 50,000 paired reads increments) from a fastq file originating from a library sequenced at high depth (Mc-B, Additional file 6). The code to reproduce this figure is available (https://github.com/rguerillot/RM-seq) and can be used to optimise sequencing depth according to the desired number of consensus reads.
